# Anosognosia in schizophrenia

**DOI:** 10.1017/S1092852924002323

**Published:** 2024-12-26

**Authors:** Benjamin Rose, Philip D. Harvey

**Affiliations:** 1 Department of State Hospitals, California; 2 Department of Psychiatry and Behavioral Sciences, University of Miami Miller School of Medicine, Miami, FL, USA

**Keywords:** anosognosia, schizophrenia, insight, Introspective Accuracy, Introspective Bias, cognition

## Abstract

Anosognosia, defined as a lack of knowledge of the disease, was originally identified in neurological disorders and is common in schizophrenia. These deficits are commonly referred to as “lack of insight” or “unawareness of illness.” They include challenges in accurate judgments of the reality of experience, as well as global and specific personal abilities. Related to inaccuracies in self-assessment are response biases when an incorrect self-assessment is made. We adopted a perspective focused on Introspective Accuracy (IA) and Introspective Bias (IB). IA is the ability to accurately judge several domains of experience and functioning. These include the reality of clinical symptoms, the experience of mood states, momentary competence in the performance of cognitive assessments and everyday functional skills, and the ability to accurately anticipate the success of future performance. IB is the direction of response bias in the context of impairments in IA. Deficits in insight, judgment inaccuracies, and response bias are highly relevant as these difficulties come with downstream impacts including difficulties with treatment adherence, an increase in severity of symptoms, greater everyday disability, reduced response to cognitive training interventions, and a need for increased intensity of interventions to maintain community residence. In this article, we review the research in IA and IB in schizophrenia, including differences in momentary versus global self-assessments, and the clinical correlates and functional impacts of inaccurate self-assessments and response biases in the context of self-assessment errors. We also examine the existing data regarding the neurobiological basis of impairments in IA.

## Introduction

As described in a thorough review of the history of Anosognosia,^1^ the neurologist Babinski coined the term anosognosia in 1914. The word comes from “a” without, “nosos” disease, and “gnosis” knowledge, or without knowledge of the disease. While getting most of the credit, Babinski was not the only researcher to study this lack of awareness, which is present in neurological and neuropsychiatric conditions. Before Babinski, the concept was defined by another neuropsychiatrist, Gabriel Anton in 1899, who considered this lack of awareness as potentially distinct from underlying neurological condition. A few years before Anton, Dejerine, Van Monakow, and Vialet systematically described the lack of awareness of disease in certain neurological conditions. Two thousand years earlier, the philosopher Seneca, sending a letter to Lucilius, had described anosognosia and captured the separation of impairment (possible brain blindness originating from a stroke) and the lack of awareness of the impairments.

*This foolish woman suddenly lost her sight … Incredible as it may appear … she does not know she is blind.*

*-Seneca*

Since the early 20th century, anosognosia, or “without knowledge of disease” has been recognized as an important component of serious mental illness. Despite the recognition of the importance of anosognosia in schizophrenia, little new knowledge was obtained throughout the first half of the 20th century. During this time the lack of treatment for psychotic symptoms made differentiation of symptoms and insight challenging. After the introduction of antipsychotic treatments in the 1950s, a shift started in the 1970s, when the World Health Organization concluded that “lack of insight,” unawareness of the implausibility of psychotic experiences, was a feature that was the most common sign of the illness.^2^ ^,^ ^3^ A few years later, there was a significant growth in interest and publications related to defining anosognosia, relabeled as insight^4^ or unawareness,^5^ targeting clinical correlates^6^ and prognostic implications,^7^ and trying to help patients with poor insight.^8^

Definitions of insight have progressed over the last 30 years and current thought includes the concepts of cognitive insight,^9^ neurocognitive insight,^10^ and clinical insight.^4^ Cognitive insight refers to an attitudinal structure involving limits in self-reflectiveness and self-certainty, which can lead to underestimations of potential. Neurocognitive insight indexes a patient’s understanding of their functioning in various neurocognitive domains compared with objective performance. Clinical insight is the awareness that one may have a mental illness, acknowledging the reality of experiences, and acceptance of the need for treatment for the illness. Impairment in insight across domains is common in patients with schizophrenia, with estimates ranging from 50% to 98% of individuals with schizophrenia not aware that they have a mental illness.^11^ ^–^^13^ The defining features of poor awareness in schizophrenia are multi-domain inaccuracies in a patient’s self-assessment, with notable impacts on multiple elements of functional outcome.

In addition to accuracy failures in self-assessment, directional patterns of mis-estimations have been seen in schizophrenia. The tendency to overestimate abilities or underestimate task difficulty is common in schizophrenia.^14^ Bias can occur because of the failure to adequately consider externally originating information, meaning that patients with schizophrenia might miss or discount important information from the world around them.^15^ Finally, there can be difficulties in discriminating between self and other generated information.^16^ Confusion on whether something is happening internally or externally clouds a patient’s ability to accurately assess what is real. This neurological impairment has enough scientific support that it is now included in the Diagnostic and Statistical Manual of Mental Disorders (DSM-5), which lists a “lack of insight or awareness of their disorder” as an associated feature supporting the diagnosis. This feature of schizophrenia is correlated with a host of downstream impacts, including challenges in treatment adherence,^17^ increased severity of symptoms,^18^ greater everyday disability,^19^ reduced response to cognitive training interventions,^20^ and increased likelihood of more intensive interventions to sustain community residence.^21^

*To know that one knows what one knows, and to know that one doesn’t know what one doesn’t know, there lies true wisdom. — Confucius (ca. 551–479 B.C.E.)*

Awareness of illness spans far beyond the recognition that psychotic symptoms are not real experiences. There are other issues in the self-assessment of features of schizophrenia, including cognitive impairments, functional disability, and altered emotional functioning. A concept that has been introduced to describe these challenges in awareness is Introspective Accuracy (IA)^22^: the ability to judge skills, experiences, moods, and successfulness of performance accurately. Challenges in IA can be measured in terms of immediate, momentary judgments (eg, “Was that answer correct?” “How many words did I remember?”)^23^ or global self-assessments (eg, “How good am I at work, social functioning or self-care?”).^24^ Global Self Assessments also commonly have an anticipatory component, such as: “How well could you do X, Y, or Z?“^25^ Such assessments are challenging for anyone,^26^ because accurate anticipation of future performance requires recollection of past performance, including the nature of the previous task, its difficulty, and perceived level of success, and comparison of the current task with prior tasks. Even minimal errors in any of these elements of judgments can skew the accuracy of estimates of functional capabilities. In people with schizophrenia, the lack of experience with functional tasks^27^ and low rates of achievement of milestones^28^ can easily lead to biases based on lack of information.

When an incorrect self-assessment (error in IA) occurs, the second important factor is the direction of the inaccurate response. Referred to as Introspective Bias (IB), it is commonly reported that people with schizophrenia manifest a predominant positive IB^29^ as a product of the tendency to underestimate difficulty or overestimate abilities.^30^ Both IA and IB are not unique to individuals with schizophrenia, as healthy individuals also struggle with having an accurate perception of their skills and abilities, with a similarly predominant positive IB seen on the part of average or poorer performers.^31^ While this response bias causes functional challenges for all individuals, these biases are often pernicious for individuals with schizophrenia, in large part because of high levels of confidence in both momentary self-assessments of performance^30^ ^,^^32^ ^–^^34^ and global judgments of abilities.^35^ ^,^^36^

This high level of confidence in self-assessment is related to the tendencies of individuals with schizophrenia to commonly make judgments based on less information than healthy controls.^37^ This “jumping to conclusions” bias is comprised of failures to consider possible sources of information and rapidly come to judgments before healthy people would be willing. In addition to overestimation and rapid decisions, schizophrenia patients also show a “resistance to disconfirmation.”^38^ Even mildly discrepant feedback can lead healthy people to recalibrate their decisions,^39^ at least temporarily. When given external feedback suggesting misestimation or inaccuracy, schizophrenia patients show reduced ability to adjust performance, accuracy assessments, and confidence in self-assessments.^40^

There is a basic information-processing bias that underlies this process, in that schizophrenia patients show a bias to prioritize decisions based on internally generated, as compared with externally provided, information. Coltheart and Davies^41^ demonstrated that patients with schizophrenia discounted new, externally originating information that was not congruent with existing information that they believed. When evaluating the reason for this discounting, Moritz et al. ^42^ suggested that external information is ignored because of challenges in problem-solving and prioritization. An additional possibility is more fundamental. Harvey et al. ^43^ showed that people with schizophrenia manifested a greater recall benefit for self-generated, as compared with externally originating, information compared with healthy individuals. Further, a well-supported hypothesis, “hyperfocusing” suggests that people with schizophrenia manifest a reduced ability to consider all elements of a complex situation because of reductions in their overall information processing capacity ^44^. Thus, when having to select information to process, they tend to focus on a limited set of more easily processed data. The universe of more easily processed information includes a predominance of self-generated information and long-held beliefs and opinions, requiring less cognitive capacity and effort to retrieve and utilize in decision-making.

### Neurological origins of anosognosia in schizophrenia

Flashman et al.^45^ noted neuroanatomical and neuropsychological similarities between anosognosia in neurological conditions and a lack of insight in schizophrenia. In neurological conditions, lesions in the frontal and right parietal lobe were observed in patients with anosognosia, and within schizophrenia, patients with poor insight have been shown to do poorly in neuropsychological assessment measures that test the frontal and parietal lobes.^46^ ^–^^48^ Shad et al.^49^ concluded that there is a similarity between anosognosia in neurological conditions and insight deficits in schizophrenia.

Neuropsychological tests have limits for identifying regionalized deficits in schizophrenia because of the background challenges of global performance deficits inducing deficits across tests of many different domains in neurological patients.^50^ To develop an integrated perspective on insight and structural and functional factors, researchers have used Voxel-Based Morphometry (VBM) to image the brain and fMRI studies to measure brain activation in schizophrenia patients across levels of insight. Structural neuroimaging has shown abnormalities in several areas of the brain for patients with schizophrenia and insight difficulties, but primarily in the prefrontal and insular cortices.^51^ Interestingly, in healthy people^52^ self-assessment ability is correlated with better functioning in both of these regions, as well as the right rostrolateral prefrontal cortex (RRPFC). Altered functioning in these areas could reduce a person’s ability to process and maintain external information (ie, working memory) while making decisions regarding its source and importance (ie, executive functioning).

Finally, researchers have considered differences in brain activation associated with IA performance using fMRI. One study in this area was conducted by Pinkham et al.^53^ In this study, participants with schizophrenia and healthy controls were imaged while they performed social cognitive tests, with and without the requirement to concurrently make an accurate judgment regarding their performance. In previous studies of momentary IA judgments, two critical frontal lobe regions, the RRPFC and dorso-anterior cingulate cortex (DACC), were found to be implicated in performance. Three levels of evidence implicating regional brain dysfunction and unawareness came out of the study. First, when compared with healthy controls, individuals with schizophrenia showed reduced activation of the RRPFC and DACC while performing the self-assessment test. Second, in healthy individuals, but not participants with schizophrenia, the level of activation of these brain regions predicted the quality of performance on the self-assessment task. Third, and critical for real-world functional implications, in participants with schizophrenia, despite the general failure to activate the brain regions during self-assessment, greater activation of the RRPFC and DACC during self-assessment were associated with independently generated ratings of social functioning. Thus, the findings regarding alterations in functioning in critical brain regions translate to real-world social functioning, which has been shown to be strongly correlated with social cognitive IA deficits and a positive IB when errors are made in social cognitive judgments.

### Clinical correlates and manifestations of IA

Challenges in IA and directional biases in IB are found for both momentary and global decision-making regarding the correctness of cognitive and functional performance and emotional processing. We advance the novel, but clearly empirically supported idea that many responses that are designated as self-assessments of immediate performance or global abilities, are actually not. In many different decisional domains responses should originate from the decision-making processes, described above, regarding recollection of past performance and comparison of the current task with prior tasks; the observable data suggests that this is not common. There is also clear evidence that participants with schizophrenia may apply different decision rules and standards for evidence compared with what would be considered normative.

### Clinical insight and treatment failures

Psychotropic medications are the first-line treatment for patients with schizophrenia. To be effective, patients must obtain the medication, take the medication as directed, and be regularly monitored by a mental health professional. Prescription medication schedules include patients taking pills daily or less frequent injectable depot medication. Effectively treating a serious mental illness is complicated, even for a motivated recipient. A lack of insight adds additional difficulty.

There are several domains where reduced insight can reduce adherence to medication regimens and impact general orientation toward treatment. Table 1 describes adherence challenges and likely origins. Reduced adherence has several consequences, including full relapse of psychotic symptoms or a chronic state of partial medication response. As described below, breakthrough psychotic symptoms appear to covary with a number of other challenges in insight and awareness, with the causal direction not exactly clear. However, there are significant risks to CNS integrity associated with non-adherence, relapse, and chronic psychotic symptoms. In a review of 13 meta-analyses and 25 657 patients, Howes et al.^54^ reported “highly suggestive” evidence that untreated psychosis leads to more severe positive symptoms, more severe negative symptoms, and lower chance of remission. Zoghbi et al.^55^ also reviewed 83 studies that looked at the neuroanatomy of untreated psychosis and concluded that while there was not sufficient evidence of global neurological impairment, specific brain structures like the temporal lobe could be vulnerable to adverse impacts of early untreated psychosis. A classic study, predating the availability of clozapine in the United States, shows these same adverse impacts in chronic patients. Davis et al.^56^ reported that chronic treatment-refractory patients showed evidence of 4-year longitudinal ventricular enlargement, particularly left-sided, compared with similarly treated chronic patients whose symptoms responded to treatment. These data underscore the importance of medication treatment and adherence to stave off avoidable neurological deterioration associated with untreated symptoms.

**Table 1.** Elements of Poor Adherence to Treatment and Possible Causes

Adherence challenge	Possible origin
Not self-administering medication	Lack of perceived need for treatment
Not attending psychiatric appointments	Lack of perceived need for treatment
Failing to refill medication	Organizational/Executive Functioning Challenges Leading to Poor Planning
Incorrectly Self-Administering Medication	Lack of understanding of the actual effects of medication/Executive Functioning
	Challenges leading to poor organization
Not taking care of physical health	Lack of understanding/Sedentary behavior Associated with Avolition

### Self-generated information and quality of test performance

In a study that sought to specifically elucidate the nature and consequences of reliance on externally provided versus self-generated information, Tercero et al.^36^ used a modified version of the Wisconsin Card Sorting Task (WCST) that included a meta-cognitive component. Participants were asked to sort 64 cards, generate an immediate accuracy judgment after each sort, and then rate their confidence in each accuracy judgment. They were then provided feedback as in the standard administration of the WCST. Participants with schizophrenia underperformed participants with bipolar disorder on sorting, while both equivalently overestimated the accuracy of their sorting performance on a momentary basis. Participants with schizophrenia were more confident in the correctness of their sorts on a momentary, sort-by-sort basis, and their confidence did not change after receiving feedback regarding sorting errors,^57^ while participants with bipolar disorder manifested confidence ratings that tracked performance feedback. However, the fundamental difference between the groups is that participants with schizophrenia based their overall judgments regarding task performance solely on their own accuracy decisions, with no correlation between feedback received (tracking the actual accuracy of their responses) and global judgments. Participants with bipolar disorder provided global judgments of their performance that were highly correlated with performance feedback.

WCST performance on the part of participants with schizophrenia was negatively influenced by reliance on recollection of self-generated information. This response pattern was also marked by the exceptional accuracy of participants with schizophrenia in recalling their self-generated responses while showing no evidence of considering the feedback they received. In this case, recalling only self-generated information rendered feedback irrelevant. The commonly observed lack of correlation between true performance and judgments of performance in participants with schizophrenia seems not to reflect random responses. Instead, it appears to be the result of applying very well-remembered information (Trial x trial responses) to the wrong purpose: recalling and using external information about performance to generate a global competence assessment.

### Momentary monitoring failures and psychotic symptoms

In a study of immediate self-assessment of cognitive performance, study participants completed an ecological momentary assessment (EMA) study with 90 surveys over 30 days. Each survey had queries about several different illness features, including the momentary occurrence and severity of psychotic symptoms. At the endpoint, a cognitive assessment was performed and participants with schizophrenia underperformed participants with bipolar disorder on five different neuropsychological tests ^23^. Participants were asked to make quantitative judgments about their performance immediately after they completed each of the five tests. On an absolute basis, mis-estimation errors had an effect size of Cohen’s *d* = 0.85 for both samples, although the directional nature of IB was different. Participants with schizophrenia overestimated their performance when they made an incorrect decision and participants with bipolar disorder underestimated. Introspective inaccuracy in schizophrenia was significantly correlated with the frequency of momentary psychotic symptoms, consistent with earlier theories of the origins of delusions as being challenges in self-assessment. However, there was no correlation between momentary psychotic symptoms and test performance, suggesting that psychosis has a greater impact on evaluating the quality of performance than on cognitive performance itself.

### Positive symptoms and impossible self-assessments

In a related challenge, people with schizophrenia may generate reports of engagement in activities that are impossible, because the requisite congruence between reports of performing the activities and their actual observed behavior are incompatible. For example, in the study mentioned above, participants with schizophrenia who were alone for over 90% of 90 EMA surveys over 30 days reported that they were more capable of engaging in activities that cannot be performed at home, including taking public transportation, in-person banking, and in-store shopping, compared with participants who were more commonly away from home with others.^58^ Interestingly the same participants who were alone were found to be more commonly experiencing psychotic symptoms on a momentary basis. Thus, the boundary between decision rules about behavior (eg, “Did I do it?” vs. “Could I do it?”) overlaps with psychotic experiences related to the lack of clinical insight. We suggest that self-reports of competence are actually ideas that originated from the same processes that lead to the development of other delusions. These impossible reports may reflect being confused about whether or not previous events actually occurred. Reporting engagement in away-from-home activities while continuously at home is not qualitatively different than reporting attempts at persecution by others that never occurred. Another source of impossible reports is described below, the application of uniquely, and idiosyncratically, applied decision rules where the boundary between recollection of actual performance and self-assessments of competence disappears.

### Are there uniquely and individually applied rules of evidence?

The process of endorsement of high competence in activities that are never performed may also originate from uniquely applied decision rules combined with response biases. These “home-alone” participants could not be making a self-assessment based on recollection of experiences (ie, success, task completion, repetition of strategies) associated with a history of performance. We have previously hypothesized that these participants are reporting on their belief that they could perform the tasks, even if they never do, and are providing an honest report on their perceived competence and not a recollection-based report of their previous behavior. Thus, a positive IB would lead to reports of engaging in activities that never occurred, wherein participants believe that they could succeed if they were given the opportunity or were somehow required to do the task.

A related phenomenon occurs with self-reports of medication adherence. There is a common, yet unusual, phenomenon related to awareness of and reports of medication adherence. People with schizophrenia may report that they are self-administering their medication when they have medication on hand but are not taking it, even when they are being tested for medication adherence. These findings suggest that they might have different definitions of what “taking medication” means, with responses based on medication possession rather than medication self-administration. Thus, the report of high levels of medication adherence would be subjectively supported by 100% medication possession, even if self-administration was negligible. Table 2 provides a heuristic model of altered decision-making as applied to oneself in a situation where implausible endorsements are generated. Consistent with jumping to conclusions regarding task-based decisions, available evidence regarding behaviors in functional situations seems to be only partially evaluated before the generation of reports of engaging in the activity. Thus, consistent with hyperfocusing, liberal acceptance bias, and a preference for self-generated information, reports of competence in engaging in activities seem to be made without considering information regarding prior performance. A self-assessment of competence, likely overestimated because of lack of experience, is reported as equivalent to success while engaging in the activity.

**Table 2.** Model of “Alternative Reality” in Reports of Engagement in Activities

Statement	Underlying logic
If I have medication at home, that means I am taking my medication	Medication adherence is redefined as medication possession because I could take it if I wanted to
If I think that I know how to take the bus, even if I have never done it, I am successful in taking the bus.	Subjective knowledge is redefined as real-world performance
I have the skills to be working at least part-time (even though the participant has never worked)	I know lots of people who are working, and I am as good at work skills as them
I do not want to take drugs anymore; so that means that I stopped taking them.	Positive intent is a synonym for actions

### Poorer functioning associated with IA and IB

IA correlates with cognitive deficits and has been found in multiple studies to correlate with neurocognitive impairments.^59^ IA errors alone do not necessarily lead to disability nor is an IA error necessarily dysfunctional. Normal range uncertainty about the quality of performance of activities can lead to thoroughness in task completion and giving a task “the once over” such as in proofreading or reviewing a shopping list, which can lead to better functioning. In such a case, the individuals might very well be responding to a mild but adaptive negative IB: “I am not sure that I have done the job thoroughly.”

IB may lead to multiple motivational challenges that have the potential to impact functioning differently depending on the direction of the bias. A positive IB, the belief that one is more competent than one actually is, can lead to reduced interest in rehabilitation-focused interventions and missing out on opportunities to learn new skills, obtain employment, or live independently. “I don’t need any help; I could get a job if I wanted to.”

Negative IB can suppress efforts to undertake tasks that are in fact possible to accomplish. IB, in either direction, does not seem to be related to poorer momentary performance on formal cognitive assessments across multiple studies.^60^ However, the lifelong experience of lower intelligence or academic ability seems to be predictive of overconfidence in healthy adults^31^ and this may also apply to people with schizophrenia.^59^ ^,^^60^

### Treatment of self-assessment challenges

Different treatments may be required for IA and IB. In theory, computerized cognitive training could improve neurocognition and thereby reduce mis-estimation, which may improve IA and reduce opportunities for the influence of IB. Response bias may be addressed by social cognitive interventions and metacognitive training. Further, a host of cognitive behavior therapies and metacognitive therapies target IB in its various forms^8^, with strategies not dissimilar to those applied to treatment-resistant delusional beliefs. Describing these interventions is beyond our scope, but these interventions are widely available and utilize a theoretical framework focusing on teaching the participant to recognize the possibility of IA and then addressing the dominant tendency toward positive IB.

Successful treatment of psychotic symptoms can lead to changes in clinical insight. It is very common for individuals whose psychosis has been remitted or even reduced through antipsychotic treatments to state that they no longer believe that their psychotic experiences were real. Improvement in psychotic symptoms has been reported to precede improvements in insight and participants with remission of psychosis at 6 months show improvements in awareness that continue to accelerate for up to 12 months.^61^ Information is harder to obtain regarding the time course of relapse, including the timing of medication discontinuation, changes in insight, and relapse. Studies have suggested that poor therapeutic alliance, negative attitudes toward medication, and substance use may lead to medication discontinuation, which then leads to changes in symptoms, which then leads to reduced insight.^17^ Further, in those studies, poor treatment response and never developing insight in response to clinical changes are also risk factors for relapse. Newly developing digital strategies may both be able to untangle the sequence of events leading up to relapse as well as provide intervention strategies.^62^

### Social-cognitive deficits as a treatment target

Social-cognitive deficits are another target for treatment. Attributional bias is common in patients with paranoia.^63^ These biases often lead to patients inferring negative intentions to others and sometimes feeling the need to engage in various actions based on goals of self-preservation. Positive IB was found to be correlated with both poor performance on social-cognitive tests^19^ ^,^^30^ ^,^^33^ ^,^^64^ and poorer social outcomes.^65^ The connection between these two impaired domains, social cognition, and social outcomes, can be related to patients overestimating the extent to which they understand other people’s intentions and interest in them. If social competence is poor, but an individual thinks that it is excellent, or even perfect, then that person might have an increased likelihood of negative interactions with others based on misperceptions, leading to confusion. Such a combination of overconfidence in abilities and misperception of others’ interest in them is likely one cause of stalking behavior on the part of people with schizophrenia.^66^

As described above, participants with schizophrenia manifesting anosognosia show deficits in the strategies they use to gather information, the amount of information that they gather, and the rapidity of decision-making, all of which also can lead to impairments in the processes referred to as a theory of mind.^67^ These social cognitive deficits have a substantial functional impact on patients as described above and can lead both to negative interactions, including violence^68^ and self-selected social avoidance^69^ based on failed attempts to interact.

Treatment of social cognitive deficits has commonly focused on interaction-focused training. Social Cognition Interaction Training or SCIT^70^ is a group treatment aimed at improving social cognitive processes for patients with psychotic disorders. SCIT looks to improve common social cognitive problems seen in schizophrenia: emotion perception, theory of mind, hostile attribution bias, and jumping to conclusions with elevated confidence levels in their beliefs. SCIT uses principles of neurocognitive remediation with Cognitive Behavioral Therapy techniques to address these deficits and biases. Multiple studies have shown that SCIT is portable across cultures and settings. Within a forensic environment,^71^ patients receiving active treatment showed statistically significant improvements in the theory of mind, emotion perception, hostile attribution bias, cognitive flexibility, social functioning, intolerance of ambiguity, and a reduction of aggressive incidents in the SCIT group compared with the control group which received coping skills training. Conversely, Dark et al.^72^ completed a randomized controlled trial that gave SCIT to patients with schizophrenia with a “befriending” group as a control. Results showed no difference in measures of social cognition. Dark et al. noted that in their community intervention, they believed that one of the difficulties was session adherence which was lower than previously published studies. An inpatient environment might be a more appropriate place for SCIT as treatment adherence can be more closely regulated. In addition, that study did not address whether patients were adherent to their psychotropic medications and did not use any standardized measures of symptoms. It is unclear the impact that psychiatric symptoms have on the patient’s ability to engage with the material in the treatment group.

Treatments for social cognitive deficits have been shown to be effective in improving domains of social cognition,^73^ but a follow-up meta-analysis suggested that improvements in social functioning are not uniformly found.^74^ Social cognition treatments require that a patient has at least a rudimentary awareness of their disorder. Again, in an inpatient environment with improved treatment adherence and developing clinical insight, social-cognitive deficits are a viable treatment target.

## Conclusions

Anosognosia is a common feature of schizophrenia. Many of the symptoms of schizophrenia directly arise from challenges in evaluating the validity and plausibility of certain experiences. Impairments in the accuracy of self-assessment appear to be related to part of the larger picture of cognitive deficits in schizophrenia, while overt biases seem to be a common and central feature of self-assessment in schizophrenia. The combination of IA/IB and possible decision-making that involves an alternative reality, can lead to self-reports that are truly uninformative when attempting to make a judgment regarding functioning.

Research has suggested dysfunctions in several different brain regions being implicated in various forms of IA and IB deficits. General awareness seems related to dysfunctions in the prefrontal cortex and specific brain structures are implicated in success in momentary judgments regarding the accuracy of socially relevant decisions. Better functioning in these regions is associated with better social outcomes, suggesting that the ability to activate critical brain regions “on demand” to make socially relevant self-assessments leads to better social outcomes.

The implications of anosognosia are broad and certain treatments (ie, antipsychotic medications) that are commonly avoided by people with schizophrenia because of impairments in clinical insight may actually hold the promise for the first inroads into impairments in awareness. Treatment of IB requires targeting a variety of attitudes related to perceptions of abilities and past achievements. The eventual goal of these treatments would be realistic self-assessment, which we have shown to have the immediate potential to improve functioning in several different domains. Clinical stability induced by medication adherence does not seem to have a direct impact on cognitive performance,^75^ but recent studies suggest that clinical stability may be a prerequisite for training gains in learning-based therapies.^76^ As noted above, sustained psychotic symptoms appear to have the potential to lead to wide-ranging deterioration in brain functioning.

Naturally, value judgments are embedded in considering evaluating refusal of treatments associated with anosognosia. On the one hand, refusing treatment for illness, even if it is potentially terminal, is generally accepted in the United States. On the other hand, anosognosia adds an extra layer of complexity to the decision-making process because individuals are not aware of their illness. Would an individual choose to have the illness treated if they did not have anosognosia? It is further complicated when a refusal to accept treatment leads to risk for others or the patient themselves. Thus, anosognosia presents a complex dilemma. Would your life be better if you accepted treatment or is refusal, despite possessing the previously mentioned “without knowledge of the disease,” simply a fundamental right of self-determination? Obviously, as treatments targeting anosognosia are further developed, it is our hope that this discussion is actually not required.
